# Computed tomography during initial management and mortality among hemodynamically unstable blunt trauma patients: a nationwide retrospective cohort study

**DOI:** 10.1186/s13049-017-0396-7

**Published:** 2017-07-19

**Authors:** Yusuke Tsutsumi, Shingo Fukuma, Asuka Tsuchiya, Tatsuyoshi Ikenoue, Yosuke Yamamoto, Sayaka Shimizu, Miho Kimachi, Shunichi Fukuhara

**Affiliations:** 1 Department of Healthcare Epidemiology, Graduate School of Medicine and Public Health, Kyoto University, Yoshida-Konoe-cho, Sakyo-ku, Kyoto 606-8501 Japan; 2 Department of Clinical Epidemiology and Health Economics, School of Public Health, Graduate School of Medicine, The University of Tokyo, 7-3-1 Hongo, Bunkyo-ku, Tokyo 113-0033 Japan; 3 Department of Emergency Medicine, National Hospital Organization Mito Medical Center, 280 Sakuranosato Ibaraki-machi, Higashiibaraki-gun, Ibaraki 311-3117 Japan

**Keywords:** Tomography, X-Ray computed, Multiple trauma, Advanced trauma life support care/SN, Shock, Mortality, Prognosis

## Abstract

**Background:**

Although many hemodynamically unstable trauma patients undergo computed tomography (CT) to identify a source of bleeding, this practice is currently only recommended by a few guidelines. To clarify whether CT has harmful effects among these patients, we examined the association between CT during initial management and mortality among unstable blunt trauma patients.

**Methods:**

This was a retrospective cohort study based on Japan Trauma Data Bank 2004–2014 registry data. Study population was adult blunt trauma patients with hypotension on arrival. The primary outcome was the in-hospital mortality. Two types of analyses were performed to adjust for confounding factors including propensity score inverse probability of treatment weighted (IPTW) and instrumental variable (IV) analysis.

**Results:**

Among 5,809 patients who met inclusion criteria, 5,352 (92.1%) underwent CT. The No CT group was more likely to have severe physiological conditions and lower probability of survival than those of the CT group. In IPTW analysis adjusting for measured confounders, we found a significant protective effect of undergoing CT on in-hospital mortality (excess deaths: −20.6 per 100 patients, 95% CI −26.2 to −14.9). In IV analysis adjusting both for measured and unmeasured confounders, the association between CT and mortality was not statistically significant (excess deaths: −4.1 per 100 patients, 95% CI −23.1 to 14.8).

**Discussion:**

We did not find clinically meaningful harmful effect of CT on survival for unstable blunt trauma patients even after adjusting both for measured and unmeasured confounders.

**Conclusions:**

Our results did not support the recommendation of current guideline. We suggest physicians should consider CT as one of the diagnostic options even when patients are unstable.

**Electronic supplementary material:**

The online version of this article (doi:10.1186/s13049-017-0396-7) contains supplementary material, which is available to authorized users.

## Background

Recently, computed tomography (CT) has become a standard initial diagnostic procedure in the management of severe trauma [1–4]. Accordingly, physicians sometimes perform CT on hemodynamically unstable patients, especially for blunt trauma cases, in order to identify a source of bleeding and assess for occult internal injury. However, CT has not been recommended for hemodynamically unstable trauma patients by most clinical guidelines [5–7]. Some have even called CT the “tunnel of death” for unstable patients [8].

Conventionally, examining these patients with CT has not been recommended according to the following reasons; 1) transporting patients to the CT scanner itself may compromise their condition, 2) resuscitation of patients in the CT room during a scan may be difficult, and 3) CT acquisition may delay definitive care.

However, evidence on the effectiveness of CT for unstable patients is inconsistent. Since 2010, one large-scale observational study has revealed the benefits of Whole-body CT (WBCT) for unstable patients [9]. In contrast, another study showed that CT was associated with a negative outcome among preoperative abdominal trauma patients [10], while other studies showed no significant association between CT and mortality [11–13]. Therefore, whether CT is really harmful for hemodynamically unstable patients remains in question.

In this study, we examined the association between CT and mortality among unstable blunt trauma patients using nationwide Japanese registry data, to clarify whether CT has harmful effect among these patients after adjusting both for measured and unmeasured confounders.

## Methods

### Study population

We performed a retrospective cohort study using Japan Trauma Data Bank (JTDB) for the years 2004–2014. The JTDB is a nationwide database of trauma patients maintained since 2004 by the Japanese Association of Trauma Surgery and the Japanese Association for Acute Medicine [14], and is one of the largest trauma registries in the world [15]. By 2014, 244 emergency hospitals voluntarily participated in this registry, most of which (81.1%) are emergency and critical centers that have equivalent role to level 1 trauma centers in the USA. These participating facilities use a Web system to collect patient data, including age and sex; vital signs on arrival, including systolic blood pressure (SBP), diastolic blood pressure (DBP), respiratory rate (RR), heart rate (HR) and Glasgow Coma Scale (GCS); type of trauma (blunt or penetrating); severity of injury using the Injury Severity Score (ISS), Revised Trauma Score (RTS) and Probability of Survival using Trauma and the Injury Severity Score (TRISS Ps) methods; CT data; and the status at discharge of each patient. Each physician enters these data without any personal information to achieve anonymization. Because we used anonymous registry data, the requirement for informed consent was waived. This study was fully approved by the ethics committee of Kyoto University School of Medicine.

### Patient selection and definitions

We included all blunt trauma patients with hypotension (SBP < 90 mmHg) on arrival. We excluded pediatric patients aged < 16 years, patients who were transferred from other facilities, and patients who suffered cardiac arrest (no heart rate; no respiratory rate; and no palpable pulse) or near-arrest (SBP ≤ 40 mmHg; based on the JTDB registration criteria that blood pressure cannot be measured at 40 mmHg SBP but a pulse is palpable) on arrival. We also excluded patients with missing data on items such as prognosis, CT information, and date of admission, as well as patients treated in facilities that had small volumes of eligible patients (<10 patients) during the study period, because we used a facility’s likelihood of performing CT as a potential instrument for instrumental variable (IV) analysis.

### The primary predictor

The primary predictor was use of CT during initial management of trauma. We defined patients undergoing CT of at least one body region as the “CT group” and patients not undergoing CT as the “No CT group.” Therefore, patients undergoing whole-body CT and those undergoing selective CT were all included in the “CT group”.

### Primary and secondary outcomes

The primary outcome of interest was in-hospital mortality. The secondary outcome was mortality within 24 h of arrival (24-h mortality). The outcome data of each patient was registered at the time of discharge or death.

### Covariates

Factors considered clinically important to the prognosis of trauma patients that can be obtained from the National Trauma Data Bank include age, ISS, gender, SBP, mechanism or type of injury, GCS, head abbreviated injury scale (AIS), abdomen AIS, race, and insurance status [16]. Among these variables, we chose age, ISS, gender, SBP, and GCS as covariates in our models. We included heart rate on hospital arrival as a covariate because it is also known to predict mortality and is commonly used as a covariate [16, 17]. We also included year of injury because the mortality of trauma patients has decreased year by year in Japan [18].

We excluded mechanisms or types of injury because our study population was restricted to blunt trauma only. We also excluded head and abdomen AIS and because these variables are difficult to measure without CT. In Japan, the public health insurance system covers all citizens and the vast majority of citizens are Asian, so we did not consider race or insurance status.

### Statistical analysis

First, we conducted a descriptive analysis of patients’ demographic data to compare the CT group to the No CT group. Continuous covariates were shown as mean (SD) and categorical covariates were expressed as numbers with percentage (%). We used Student’s *t*-test for continuous data and Pearson’s chi-squared test for categorical data for comparison. We also compared the distribution of clinically relevant covariates between the two groups.

Second, we estimated the number of excess deaths per 100 patients in the CT group compared to the No CT group as a reference by estimating the absolute risk differences of in-hospital and 24-h mortality between these two groups. We performed univariate analysis using a generalized linear model with identity link function for binomial outcomes. We also performed multivariate analysis using the inverse probability of treatment weighted (IPTW) estimator based on propensity score (PS) and IV analysis. We excluded patients with missing data for any covariate included in the model.

### IPTW method

We used the IPTW method to adjust for measured confounders [19]. We estimated PS based on clinically important variables including age, sex, vital signs at hospital arrival (SBP, HR, GCS), ISS, and year of injury. We used a logistic regression model conditionally on these variables to calculate PS. Next, we calculated the inverse probability of treatment, i.e. 1/PS for the CT group and 1/(1-PS) for the No CT group, and used these inverse probabilities of treatment as weighting factors to produce a pseudo-population where the probability of undergoing CT was equivalent. Then, we estimated the effect of CT on in-hospital and 24-h mortality using a generalized linear model with an identity link function for a binomial outcome weighted by IPTW.

### IV method

The IV method was also used to adjust for unmeasured confounders [20, 21]. An eligible instrument is a variable that is significantly associated with treatment selection and only affects patient outcome through this association (CT in this study). We chose the institutional preference of performing CT as a potential instrument. Practically, we defined our instrument as the number of the previous three admitted patients who underwent CT. This approach for defining preference by previous patients’ workup has been described previously [21, 22].

We confirmed the validity of the instrument by testing its association with our main predictor of the actual likelihood of performing CT using partial F-statistics. Partial F-statistics > 10 were regarded as valid instruments [23]. We also assumed that this instrument had no direct relation on our outcome.

We performed two-stage least squares estimation for the IV analysis. In the first-stage model, we estimated the probability of undergoing CT using least squares estimation conditional on IV and covariates including age, sex, SBP, HR, GCS on arrival, ISS, and year of injury. In the second-stage model, we estimated the effect of CT on in-hospital and 24-h mortality using a least squares estimation conditional on the probability of undergoing CT and the same covariates.

We performed sensitivity analysis using different definitions of instruments to confirm the robustness of our main analyses. Practically, we redefined our instruments as the total number of patients undergoing CT among the previous one, five, and seven patients as instruments.

Analyses were conducted using commercial software (STATA MP4 version 13.0®, StataCorp, College Station TX, USA).

## Results

### Characteristics of study subjects

**Fig. 1:**
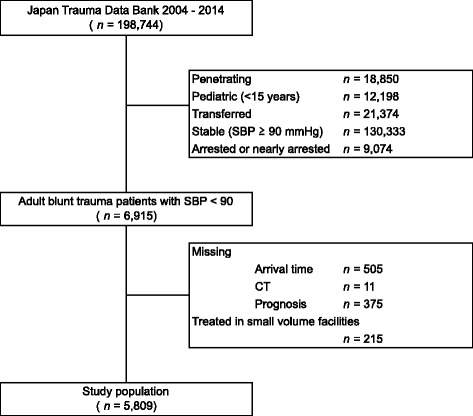
Flow chart reveals the reason for, and number of, patient exclusions

A total of 198,744 patients were registered in the JTDB between January 2004 and December 2014. Of these, 6,915 patients were eligible adult blunt trauma patients with hypotension who were directly transported from the trauma site. Of these 6,915 patients, 1,106 were excluded because of missing data or ineligibility for IV analysis due to being treated in a small volume facility (<10). Eventually, 5,809 met our inclusion criteria (Fig. 1). Summary statistics of patient baseline demographics are shown in Table 1 and Table 2. Mean age of the whole population was 56.4 years old and 65.7% were male. At hospital arrival, mean SBP was 75.6 mmHg, HR was 93.4 beats/min and GCS was 11.3. Mean ISS was 26.0 and TRISS Ps was 0.67. Overall, 5352 patients (92.1%) underwent CT (CT group). Their crude in-hospital mortality was 25.5% (Table 1). A total of 1,239 (22.4%) patients showed positive Focused Assessment Sonography for Trauma (FAST) findings. The CT group showed higher SBP, RTS, TRISS Ps, a lower proportion of positive FAST results, and a lower HR than the No CT group (Table 2). There was sufficient overlap in the distribution of these covariates and propensity scores, indicating the validity of comparing these two groups (Additional file 1: Figure S1, Additional file 2: Figure S2).

**Table 1:** Baseline characteristics of overall study population

Characteristics	Total	Missing
	(*n* = 5,809)	*n* (%)
Age, mean (SD), years	56.4 (20.7)	
Gender, male (%)	3,813 (65.7)	1 (0.02)
Year of the injury (%)		
2004	164 (2.8)	
2005	197 (3.4)	
2006	160 (2.8)	
2007	339 (5.8)	
2008	493 (8.5)	
2009	550 (9.5)	
2010	674 (11.6)	
2011	765 (13.2)	
2012	907 (15.6)	
2013	862 (14.8)	
2014	698 (12.0)	
Arrival vital signs, mean (SD)		
SBP^*a*^, mmHg	75.6 (10.1)	
DBP^*b*^, mmHg	47.0 (12.2)	952 (16.4)
RR^*c*^, breaths/min	24.0 (8.2)	563 (9.7)
HR^*d*^, beats/min	93.4 (28.0)	81 (1.4)
GCS^*e*^	11.3 (4.3)	269 (4.6)
Severity, mean (SD)		
ISS^*f*^	26.0 (15.2)	146 (2.5)
RTS^*g*^	5.87 (1.41)	740 (12.7)
TRISS Ps^*h*^	0.67 (0.31)	899 (15.5)
Underwent CT (%)	5,352 (92.1)	
FAST^*i*^, positive (%)	1,239 (22.4)	284 (4.9)
Death (%)		
In-hospital	1,483 (25.5)	
Death within 24 h	802 (14.5)^j^	

^*a*^SBP, systolic blood pressure; ^*b*^DBP, diastolic blood pressure; ^*c*^RR, respiratory rate; ^*d*^HR, heart rate; ^*e*^GCS, Glasgow Coma Scale; ^*f*^ISS, Injury Severity Score; ^*g*^RTS, Revised Trauma Score; ^*h*^TRISS Ps, Trauma Revised Injury Severity Score Probability of Survival; ^*i*^FAST, Focused Assessment Sonography for Trauma

^j^280 dead patients with missing time of death data were excluded from the numerator

**Table 2:** Baseline characteristics of the CT group and No CT group

Characteristics	CT group	No CT group	
	(*n* = 5,352)	(*n* = 457)	*p* value
Age, mean (SD), year	56.2 (21.8)	58.7 (20.6)	0.013
Gender, male (%)	3,535 (66.1)	278 (60.8)	0.024
Year of injury			
2004	148 (2.8)	16 (3.5)	<0.001
2005	172 (3.2)	25 (5.5)	
2006	140 (2.6)	20 (4.4)	
2007	301 (5.6)	38 (8.3)	
2008	437 (8.2)	56 (12.3)	
2009	516 (9.6)	34 (7.4)	
2010	622 (11.6)	52 (11.4)	
2011	712 (13.3)	53 (11.6)	
2012	852 (15.9)	55 (12.0)	
2013	802 (15.0)	60 (13.1)	
2014	650 (12.1)	48 (10.5)	
Arrival vital signs, mean (SD)			
SBP^*a*^, mmHg	75.8 (10.0)	73.6 (11.1)	<0.001
DBP^*b*^, mmHg	47.1 (12.1)	45.1 (13.7)	0.003
RR^*c*^, breaths/min	23.9 (8.2)	24.6 (8.6)	0.079
HR^*d*^, beats/min	92.9 (27.7)	99.2 (31.2)	<0.001
GCS^*e*^	11.4 (4.3)	10.9 (4.8)	0.008
Severity, mean (SD)			
ISS^*f*^	26.2 (15.1)	23.9 (16.8)	0.002
RTS^*g*^	5.89 (1.40)	5.54 (1.57)	<0.001
TRISS Ps^*h*^	0.68 (0.31)	0.64 (0.34)	0.047
FAST^*i*^, positive (%)	1,088 (21.4)^j^	151 (35.2)^j^	<0.001
Death (%)			
In-hospital	1,276 (23.8)	207 (45.3)	<0.001
Death within 24 h	655 (12.8)^k^	147 (34.9)^k^	<0.001

^*a*^SBP, systolic blood pressure; ^*b*^DBP, diastolic blood pressure; ^*c*^RR, respiratory rate; ^*d*^HR, heart rate; ^*e*^GCS, Glasgow Coma Scale; ^*f*^ISS, Injury Severity Score; ^*g*^RTS, Revised Trauma Score; ^*h*^TRISS Ps, Trauma Revised Injury Severity Score Probability of Survival; ^*i*^FAST, Focused Assessment Sonography for Trauma

^j^256 patients in the CT group and 28 patients in the No CT group with missing FAST data were excluded from the numerator

^k^244 dead patients in CT group and 36 dead patients in No CT group with missing data of death time were excluded from the numerator

### Main results

**Fig. 2:**
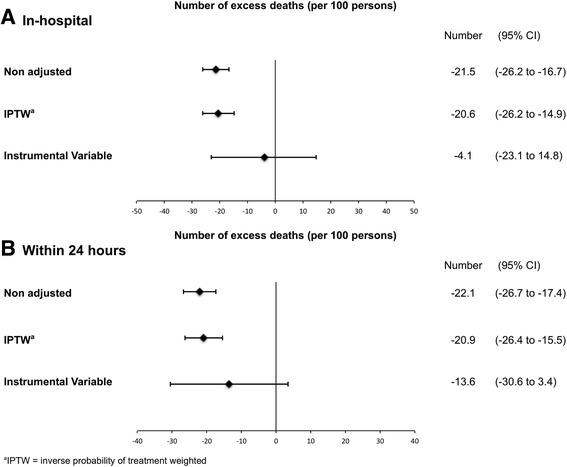
**a** Number of excess deaths per 100 patients for in-hospital mortality by non-adjusted, inverse probability of treatment weighted analysis and instrumental variable analysis. **b** Number of excess deaths per 100 patients for 24 h mortality by non-adjusted, inverse probability of treatment weighted (IPTW) analysis and instrumental variable (IV) analysis. We included age, gender, vital signs on arrival (systolic blood pressure, heart rate and Glasgow Coma Scale), Injury Severity Score and year of injury as covariate in both analyses

We performed complete case analysis, which means we excluded patients with missing covariates from the analysis. As a results, total 5,345 patients were included in IPTW analysis and 5,008 patients were included in IV analysis. Figure 2 compares the number of excess deaths per 100 patients of the two groups. Unadjusted comparison revealed that the CT group had significantly lower mortality than the No CT group. Multivariate analysis using IPTW methods also showed that the CT group had significantly lower in-hospital and 24-h mortality than the No CT group (excess deaths: −20.6 per 100 patients, 95% CI −26.2 to −14.9 for in-hospital mortality; and −20.9 per 100 patients, 95% CI −26.4 to −15.5 for 24-h mortality, respectively)

Our instruments showed a strong association with the actual performance of CT (partial F statistics 99.1 for in-hospital mortality; and 101.7 for 24-h mortality). After adjusting unmeasured confounders using the IV method, there was no significant difference between the two groups in in-hospital or 24-h mortality (excess deaths: −4.1 per 100 patients, 95% CI −23.1 to 14.8 for in-hospital mortality; and −13.6 per 100 patients, 95% CI −30.6 to 3.4 for 24-h mortality, respectively) (Fig. 2).

In sensitivity analyses, we found similar associations to the main analysis after changing the definition of our instruments (Table 3).

**Table 3:** Sensitivity analysis using differently defined instrumental variables

Instrumental Variable	Excess deaths per 100 patients	95% confidence interval	No. of patients	F statistics
In-hospital mortality				
Previous patient’s CT performance	−13.5	−40.2 to 13.3	5,230	48.8
Previous 5 patients’ CT performance	−2.4	−18.7 to 13.8	4,783	151
Previous 7 patients’ CT performance	−3.9	−19.3 to 11.4	4,548	187.5
24-h mortality				
Previous patient’s CT performance	−13.7	−37.3 to 9.9	5,000	48.6
Previous 5 patients’ CT performance	−10.2	−25.1 to 4.6	4,572	155.1
Previous 7 patients’ CT performance	−14	−28.3 to 0.0	4,349	188.5

## Discussion

In Japan, more than 90% of unstable blunt trauma patients received CT during initial management, which is not recommended in most of the current clinical guidelines. However, we did not find a clinically meaningful harmful effect of CT on survival after adjusting both for measured and unmeasured confounders by IV method. Our results do not support the recommendation of current guidelines that indicates CT should be used only for hemodynamically stable patients. We suggest physicians should consider CT as one of the diagnostic options even when patients are unstable.

Most guidelines do not recommend CT for hemodynamically unstable patients [5–7]. In Japan, the ATLS-equivalent guidelines, Japan Advanced Trauma Evaluation and Care (JATEC) [24], have been widely adopted. Physicians predominantly treat patients according to the “no CT for unstable patients” policy, similar to that indicated in the ATLS. However, in almost all hospitals in this database, multidetector CT scanners were located near or in the emergency room, allowing rapid CT for unstable patients depending on patients’ status and physicians’ preferences. In this study, 92.1% of patients underwent CT. Further, 76.1% of the CT group had negative FAST findings, which may lead physicians to avoid CT. However, some studies have suggested the low sensitivity of FAST [25, 26]: a negative FAST finding does not always indicate the absence of severe abdominal injury. For example, a patient with no abdominal complaints may have a head or chest injury characterized by a change in mental status or chest pain. In such situations, head or chest but not abdominal CT may be conducted. As a result, many patients with negative FAST findings underwent CT. Recently, the number of CT scans for trauma patients has been substantially increasing globally [27, 28]. And radiation exposure due to excessive CT use has been discussed recently [29]. Therefore, appropriate CT use for trauma patients has become important issue globally.

There has been no definitive evidence to show harmful effect of CT for unstable trauma patients. Among preoperative abdominal trauma patients, one previous study reported harmful effect of CT [10]. Another previous study reported that there was no significant difference of mortality between patients underwent CT and those not underwent CT in different population of unstable trauma patients including abdominal and non-abdominal trauma patients [13]. Those previous studies have critical limitations to examine the effect of CT in observational studies, due to unmeasured confounders.

Randomized controlled trials (RCTs) are considered the ideal method to estimate treatment effects, except for the possible effects of confounding factors. However, it is difficult to conduct RCTs among unstable trauma patients because of ethical concerns and the difficulty of recruitment of those patients. Further, observational studies have some advantages over RCTs. In this study, we could include participants who reflected the exact population irrespective of whether physicians ordered CT or not and were able to reveal the practice patterns and effect of CT in the real-world.

One of the most important problems in estimating a treatment effect in observational studies is confounding by indication, which is sometimes difficult to measure directly [30]. Clearly, the indication for CT in unstable patients is not based on random allocation but is dependent on individual patient condition and facility-level restrictions. For example, physicians tend not to order a CT if the patient is considered too unstable to tolerate the scan procedure. Physicians also cannot order CT if his or her facility does not have a CT scanner. Thus, these factors are potential confounders between CT and mortality. To resolve this problem, the IV method is a popular approach to cope with unmeasured confounders and enables the estimation of more unbiased results [21–33]. On the other hand, it has the disadvantage of a tendency towards wide 95% confidence intervals [31, 34, 35]. In our database, some clinically important predictors such as response to initial fluid resuscitation and change in vital signs after hospital arrival were not measured. We assumed the results of the IPTW method were implausible because of the likely effect of these unmeasured confounders on both CT ordering and mortality that could not be ignored. We therefore focused on the results of the IV method. Previous study supported this idea to implicate the results of the IPTW method and IV method [36].

Our study had some limitations. First, only 7.9% of participants did not undergo CT and might have represented a different population from the other 92.1%. For example, patients in the No CT group may have been more likely to have severe physiological conditions and lower probability of survival than those in the CT group. To consider this issue, we compared the distribution of major measured confounders and found that there was sufficient overlap of measured characteristics between the two groups. We therefore considered the two groups to be comparable. Second, we could not elucidate whether our instrument variable meet the criteria of an IV method based on measured data. Although it is impossible to prove there was no direct relation between our instrument and outcomes, we consider our instrument to be acceptable because most facilities included in this study were tertiary care hospitals which have no restriction on the performance of CT. Therefore, the preference to order CT was not likely related to the hospital’s quality of care. Moreover, patient-level confounders might have had a greater influence on the physician’s choice to order CT. Additionally, physicians may have underestimated the ISS in the No CT group, which had a significantly higher mortality rate. This underestimation might have had an influence on the results.

## Conclusion

In summary, most unstable blunt trauma patients undergo CT as part of initial management in Japan. We did not find clinically meaningful harmful effect of CT on survival for unstable blunt trauma patients after adjusting both for measured and unmeasured confounders. Our results do not support the current guidelines, of which only a few recommend CT for unstable patients. Continued discussion and more studies of this issue are necessary to improve the quality of care of blunt trauma patients.

## Additional files

Additional file 1: Figure S1. Distribution of measured confounders including age, systolic blood pressure (SBP), heart rate (HR), Glasgow Coma Scale (GCS), Injury Severity Score (ISS), Revised Trauma Score (RTS), and Trauma Revised Injury Severity Score Probability of Survival (TRISSPs). The distribution of these measured confounders for the CT group (solid line) and No CT group (dotted line) markedly overlapped, suggesting that these two groups were not substantially different despite the No CT group having a much lower number of patients. (TIF 26330 kb)

Additional file 2: Figure S2. The distribution of propensity scores of the CT group and No CT group. The sufficient overlap in propensity scores indicates that there were no significant differences between the two groups. (TIF 7131 kb)

## Abbreviations

AIS: Abbreviated injury scale
CT: Computed tomography
DBP: Diastolic blood pressure
GCS: Glasgow coma scale
HR: Heart rate
IPTW: Inverse probability of treatment weighted
ISS: Injury severity score
IV: Instrumental variable
JTDB: Japan trauma data bank
PS: Propensity score
RR: Respiratory rate
RTS: Revised trauma score
SBP: Systolic blood pressure
TRISS Ps: Trauma revised injury severity score probability of survival
