# Supplementary figures and images for: Computed tomography during initial management and mortality among hemodynamically unstable blunt trauma patients: a nationwide retrospective cohort study
Source: Scand J Trauma Resusc Emerg Med. 2017 Jul 19;25:74. doi: 10.1186/s13049-017-0396-7 (PMC5518106; doi:10.1186/s13049-017-0396-7)

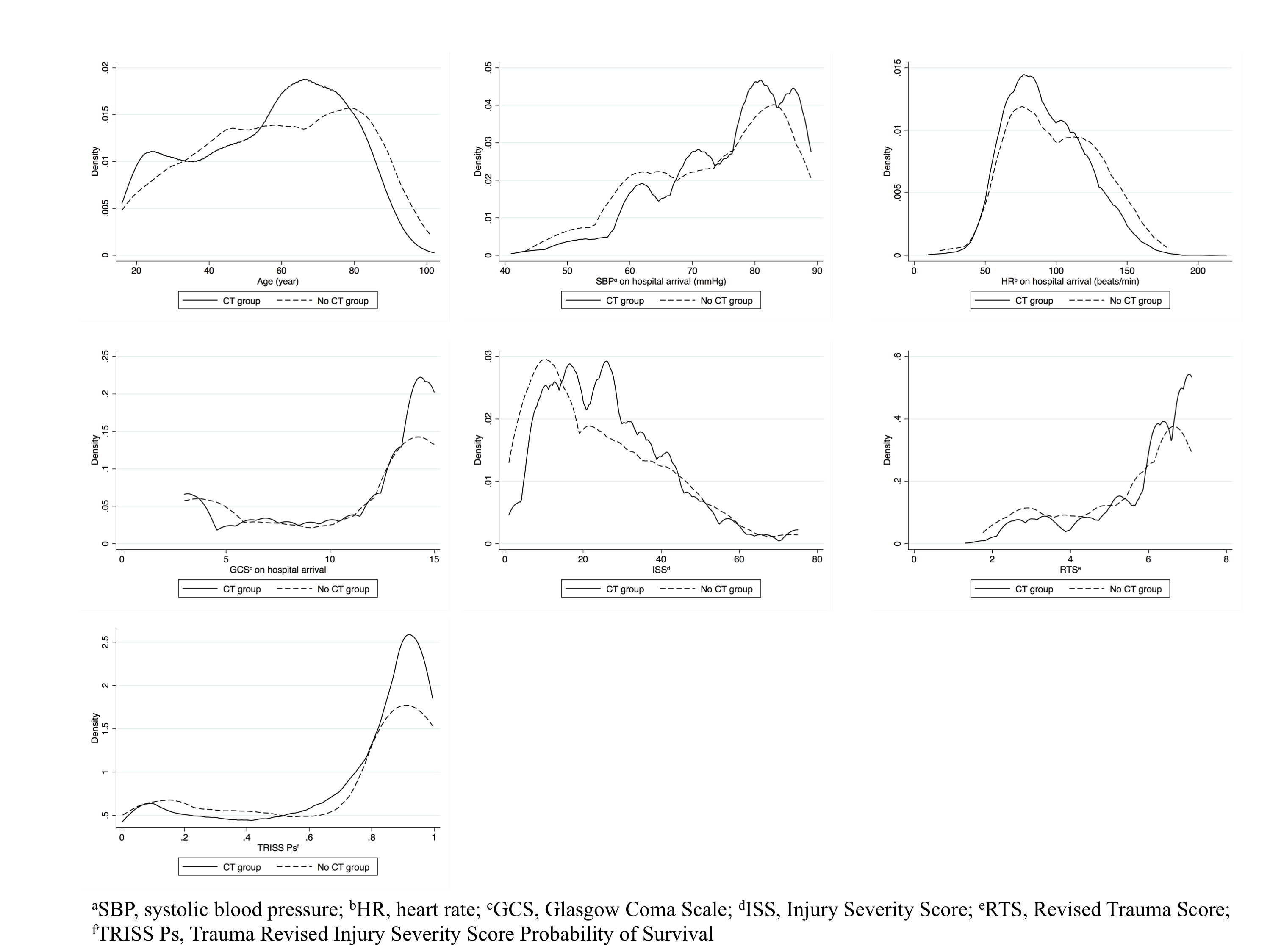

Supplement: Supplementary file 1 — Distribution of measured confounders including age, systolic blood pressure (SBP), heart rate (HR), Glasgow Coma Scale (GCS), Injury Severity Score (ISS), Revised Trauma Score (RTS), and Trauma Revised Injury Severity Score Probability of Survival (TRISSPs). The distribution of these measured confounders for the CT group (solid line) and No CT group (dotted line) markedly overlapped, suggesting that these two groups were not substantially different despite the No CT group having a much lower number of patients. (TIF 26330 kb) [file 13049_2017_396_MOESM1_ESM.tif]

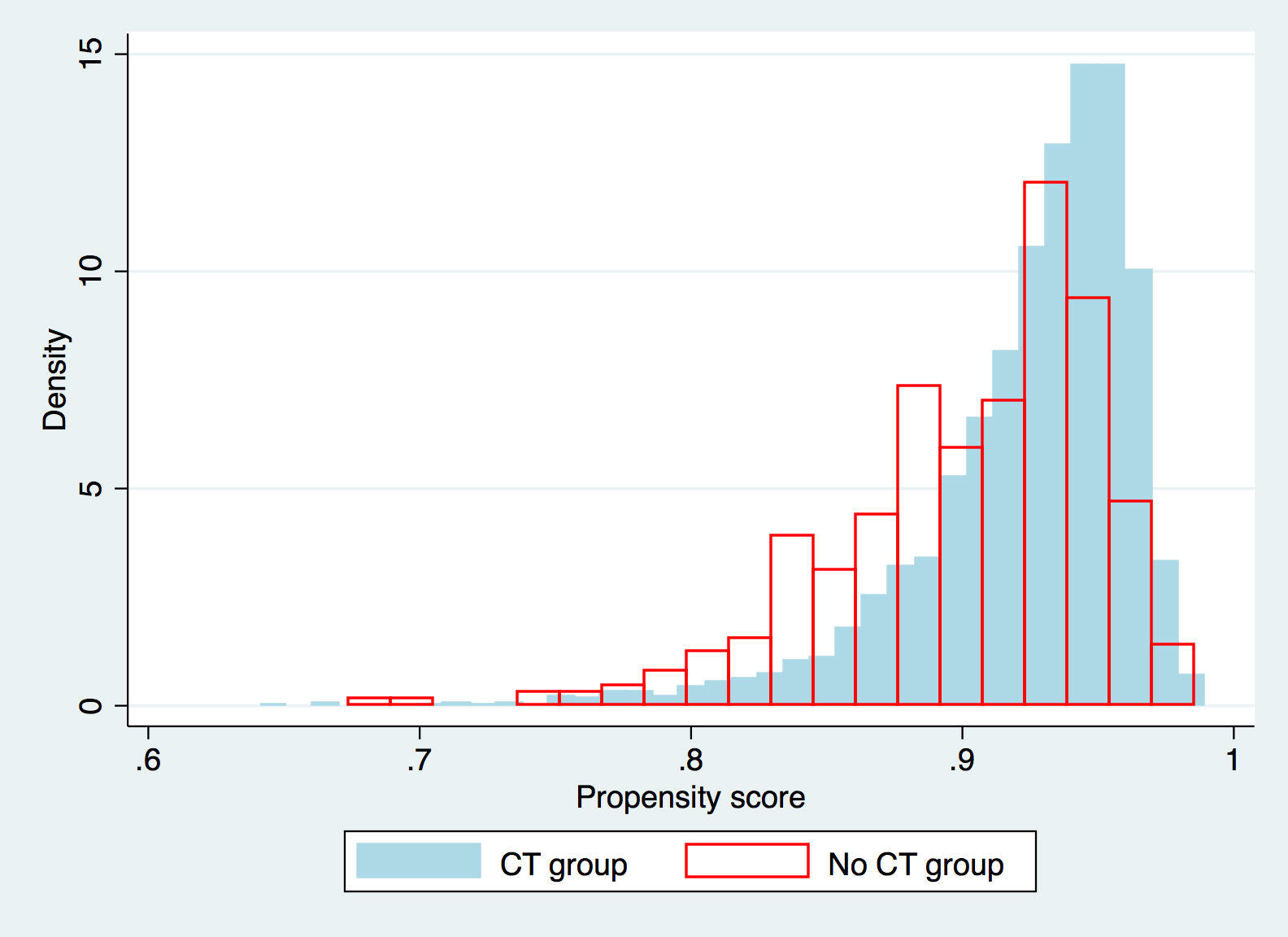

Supplement: Supplementary file 2 — The distribution of propensity scores of the CT group and No CT group. The sufficient overlap in propensity scores indicates that there were no significant differences between the two groups. (TIF 7131 kb) [file 13049_2017_396_MOESM2_ESM.tif]
